# Systemic regulation of L-carnitine in nutritional metabolism in zebrafish, *Danio rerio*

**DOI:** 10.1038/srep40815

**Published:** 2017-01-19

**Authors:** Jia-Min Li, Ling-Yu Li, Xun Qin, Li-Jun Ning, Dong-Liang Lu, Dong-Liang Li, Mei-Ling Zhang, Xin Wang, Zhen-Yu Du

**Affiliations:** 1 Laboratory of Aquaculture Nutrition and Environmental Health (LANEH), School of Life Sciences, East China Normal University, Shanghai, China; 2 Shanghai Key Laboratory of Regulatory Biology, Institute of Biomedical Sciences and School of Life Sciences, East China Normal University, Shanghai, China

## Abstract

Excess fat accumulation has been observed widely in farmed fish; therefore, efficient lipid-lowering factors have obtained high attention in the current fish nutrition studies. Dietary L-carnitine can increase fatty acid β-oxidation in mammals, but has produced contradictory results in different fish species. To date, the mechanisms of metabolic regulation of L-carnitine in fish have not been fully determined. The present study used zebrafish to investigate the systemic regulation of nutrient metabolism by dietary L-carnitine supplementation. L-carnitine significantly decreased the lipid content in liver and muscle, accompanied by increased concentrations of total and free carnitine in tissues. Meanwhile, L-carnitine enhanced mitochondrial β-oxidation activities and the expression of carnitine palmitoyltransferase 1 mRNA significantly, whereas it depressed the mRNA expression of adipogenesis-related genes. In addition, L-carnitine caused higher glycogen deposition in the fasting state, and increased and decreased the mRNA expressions of gluconeogenesis-related and glycolysis-related genes, respectively. L-carnitine also increased the hepatic expression of mTOR in the feeding state. Taken together, dietary L-carnitine supplementation decreased lipid deposition by increasing mitochondrial fatty acid β-oxidation, and is likely to promote protein synthesis. However, the L-carnitine-enhanced lipid catabolism would cause a decrease in glucose utilization. Therefore, L-carnitine has comprehensive effects on nutrient metabolism in fish.

L-carnitine is an important factor that regulates fatty acid (FA) and glucose metabolism to achieve balanced cellular energy metabolism^1^. L-carnitine can be synthesized naturally from lysine and methionine in microorganisms, plants and animals^2^. L-carnitine plays a vital role in the β-oxidation of long chain fatty acids (LCFAs) to produce energy by transporting LCFAs from the cytosol into the mitochondrial matrix, and can modulate the ratio of acetyl-CoA/CoA^3^,^4^. In humans and other mammals, L-carnitine deficiency causes severe hyperlipidaemia and systemic metabolic syndrome^5^,^6^. Accordingly, dietary supplementation of L-carnitine had been reported widely to improve lipid metabolism and/or alleviate the high fat diet (HFD)-associated excess lipid accumulation in humans and other land animals^7^,^8^,^9^.

Compared with other farmed animals, fish generally have a lower ability to use carbohydrates as energy sources; thus requiring higher levels of dietary proteins^10^. With the increasing cost and limited supplies of fish feed protein, HFDs are used currently in aquaculture for their “protein sparing effect”, which means that the energy that is normally sourced from protein consumption is replaced by lipid-sourced energy^11^,^12^. However, HFDs often lead to severe lipid accumulation in the liver and abdominal adipose tissues in farmed fish, and induce metabolic disturbances, such as fatty liver and excess mesenteric fat deposition^12^,^13^. Therefore, regulatory factors that could reduce lipid deposition in fish, especially during HFD feeding, have attracted increased research attention. The effective lipid-lowering effects of L-carnitine have led to the hypothesis that L-carnitine in fish would have similar effects to those in mammals and would improve lipid utilization.

During recent decades, the potential nutritional functions of carnitine have been studied in many fish species; however, the results and conclusions differed. In European sea bass^14^, red sea bream^15^ and hybrid striped bass^16^, carnitine promoted growth. However, this effect was not observed in channel catfish^17^, rainbow trout^18^ and hybrid tilapia^19^. In European sea bass^14^, Mozambique tilapia^20^, Indian major carp rohu^21^, dietary carnitine supplementation could lower the lipid content in the body and liver, and it improved the utilization of certain FAs, such as eicosapentaenoic acid (EPA) and docosahexaenoic acid (DHA) in red sea bream^15^, and African catfish^22^. However, carnitine supplementation had no effects on lipid metabolism in African catfish^23^, hybrid tilapia^19^ and hybrid striped bass^16^. Indeed, carnitine even reduced lipid metabolism in red sea bream^15^, yellow catfish^24^ and rainbow trout^25^.

The contradictory and complicated results of carnitine supplementation in fish have made the further utilization of carnitine in fish feed controversial. However, compared with the intensive mechanistic studies of carnitine in mammals, the majority of the studies of carnitine in fish have only focused on the effects of carnitine on the fish’s growth and body composition, whereas studies on the systemic regulation of nutritional metabolism by carnitine in fish, and the underlying mechanisms, are lacking. This has largely restricted our understanding of the function of carnitine in fish, and an explanation of the conflicting results of the application of carnitine in fish remains elusive. Therefore, the mechanisms of the effects of carnitine in fish should be studied.

As a widespread model animal in research areas such as development, molecular genetics, toxicology and biomedicine^26^,^27^, zebrafish (*Danio rerio*) has been used in mechanistic studies of fish nutrition^28^,^29^. In the present study, zebrafish was used to evaluate the systemic regulation of lipid, protein and carbohydrate metabolism by L-carnitine in fish in feeding and fasting states, respectively. Moreover, the metabolic pathways associated with the nutritional regulation by dietary L-carnitine supplementation in zebrafish were determined. To the best of our knowledge, this is the first study to demonstrate the metabolic mechanisms of nutritional regulation by dietary L-carnitine supplementation in fish.

## Results

### The effect of dietary L-carnitine supplementation on the carnitine concentration, carnitine synthesis gene expression and triglyceride levels in tissues

**Figure 1:**
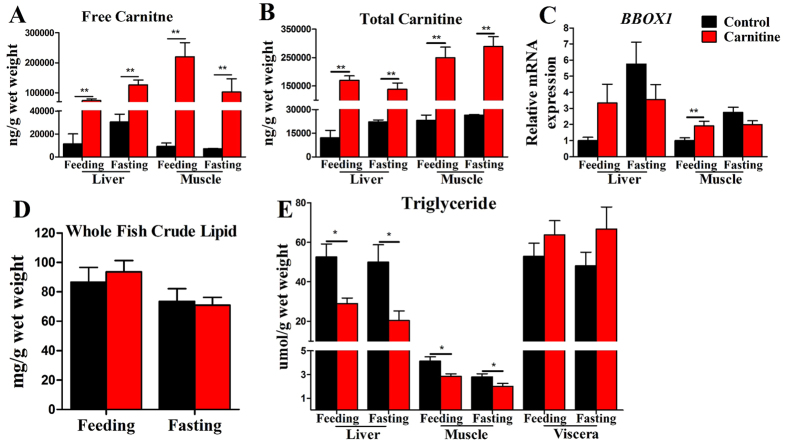
Effect of the dietary L-carnitine on the carnitine concentration, gene of carnitine synthesis and triglyceride in tissues of zebrafish. (**A**) Free carnitine; (**B**) Total canitine; (**C**) The relative mRNA abundance of carnitine synthesis (gamma-butyrobetaine hydroxylase 1, *BBOX1*); (**D**) The crude lipid content of whole fish; (**E**) TG content of liver, muscle, viscera. All values are means ± SEM (n = 6). Values with *^,^**statistically differ at *P* < 0.05, *P* < 0.01.

During the experiment, the fish were in good health and showed no growth differences compared with control fish (data not shown). To test the effects of exogenous carnitine supplementation on the endogenous carnitine concentrations, the carnitine concentrations in various fish tissues were measured. In the liver and muscle, both in the feeding and fasting states, the free carnitine and total carnitine concentrations in the carnitine supplementation group were significantly higher than in the control group (Fig. 1A,B). The mRNA levels of *BBOX1*, the key enzyme for carnitine synthesis, were not significantly different in the liver and in fasting state muscle; however, in feeding state muscle, the *BBOX1* mRNA level in the carnitine supplementation group was significantly higher than that in the control group (Fig. 1C). No significant difference was found in the whole fish lipid level in all groups (Fig. 1D). Compared with the control group, the triglyceride (TG) content was significantly decreased in the carnitine supplementation group in the liver and muscle in both the feeding and fasting states; however, the TG content in the viscera was comparable in all groups (Fig. 1E). There were no interaction effects of the nutritional states and L-carnitine found in the whole fish lipid level and TG content in the different organs, except in the *BBOX1* mRNA level (Supplemental Table 1), showing *BBOX1* is simultaneously regulated by nutritional state and L-carnitine supplementation. The above data indicated that dietary carnitine supplementation could increase the *in vivo* carnitine concentration and reduce the TG content in the liver and muscle.

### The effect of dietary L-carnitine supplementation on mitochondrial and peroxisomal β-oxidation

**Figure 2:**
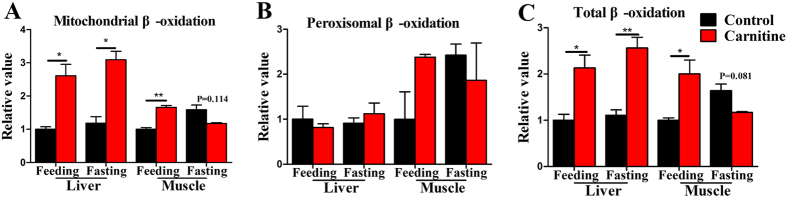
Effect of the dietary L-carnitine on mitochondrial and peroxisomal β-oxidation capability of zebrafish. (**A**) The mitochondrial β-oxidation capability; (**B**) The peroxisomal β-oxidation capability; (**C**) The total β-oxidation capability. All values are means ± SEM (n = 4). Values with *^,^**statistically differ at *P* < 0.05, *P* < 0.01.

As shown in Fig. 2, in the liver, the mitochondrial and total β-oxidation capability in carnitine supplementation group in the feeding and fasting state were significantly increased (Fig. 2A,C); however, peroxisomal β-oxidation capability was not affected. In the muscle, the mitochondrial and total β-oxidation capability in the feeding state were higher in the carnitine supplementation group than in the control, but showed no difference in the fasting state (Fig. 2A,C). There were no significant differences in the peroxisomal β-oxidation capability between the control and carnitine supplementation groups (Fig. 2B). The significant interaction between the nutritional state and L-carnitine was only seen in the mitochondrial and total β-oxidation capability in muscle (Supplemental Table 2). The results showed that dietary L-carnitine supplementation targets the liver and muscle, and has a lipid-lowing effect that functions mainly through mitochondrial FA β-oxidation, but not via peroxisomal FA β-oxidation.

### The effect of dietary L-carnitine on the mRNA expression of lipid metabolism genes

**Figure 3:**
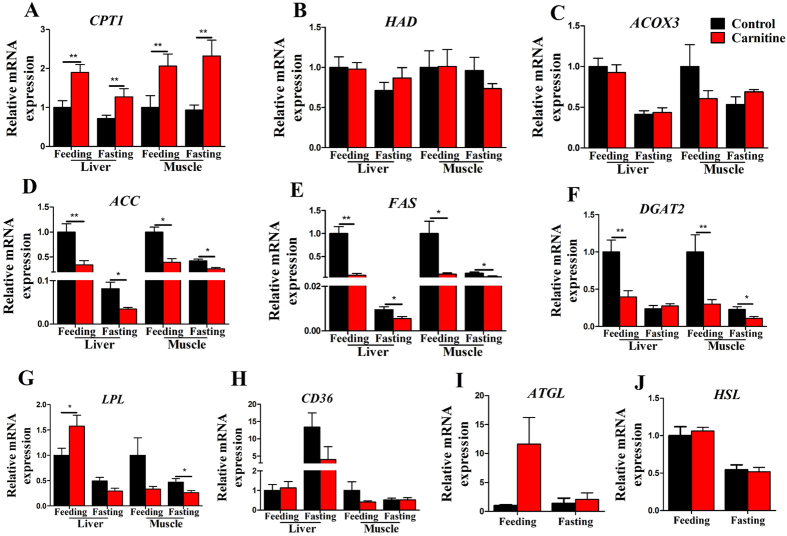
The effect of the dietary L-carnitine on mRNA expression of genes of lipid metabolism. All values are means ± SEM (n = 6). Values with *^,^**statistically differ at *P* < 0.05, *P* < 0.01.

Figure 3 shows the effects of dietary L-carnitine on the mRNA expressions of genes related to lipid metabolism. In the L-carnitine supplementation group, the mRNA level of the mitochondrial β-oxidation-related gene *CPT1* was significantly increased (Fig. 3A), whereas the mRNA levels of the adipogenesis-related genes, such as *ACC, FAS*, and *DGAT2*, were significantly decreased (Fig. 3D–F), either in the liver or muscle. The mRNA level of *LPL* increased in the carnitine supplementation group in the liver in the feeding state but decreased in the muscle in the fasting state (Fig. 3G). The other lipid metabolism-related genes, including *HAD, ACOX3, CD36, ATGL* and *HSL*, remained unchanged in the livers and muscles of the two groups in the two states. Interaction effects between the nutritional state and carnitine levels were observed in the mRNA levels of *ACC, FAS*, and *DGAT2* in liver and muscle, and *ATGL* and *LPL* in liver (Supplemental Table 3), showing nutritional state and L-carnitine both regulated the lipid synthesis and lipolysis. The results suggested that dietary L-carnitine could upregulate the mRNA expression of mitochondrial FA β-oxidation genes, and downregulate the mRNA expression of FA and TG synthesis genes in the liver and muscle.

### The effect of the dietary L-carnitine on glycogen and glucose metabolism

**Figure 4:**
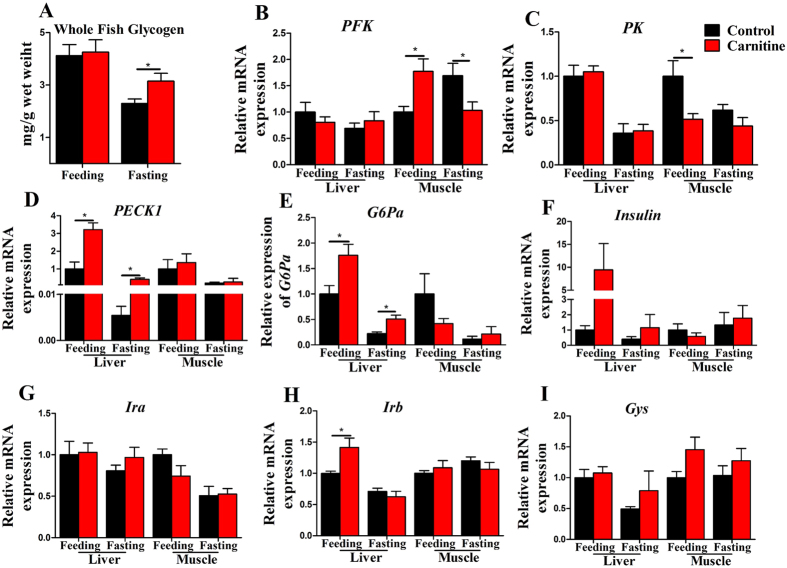
The effect of the dietary L-carnitine on glycogen and mRNA expression of the genes related to glucose metabolism. All values are means ± SEM (n = 6). Values with *^,^**statistically differ at *P* < 0.05, *P* < 0.01.

The whole fish glycogen levels remained invariant in feeding states, but increased in fasting state (Fig. 4A), and the mRNA expression of glycogen synthase (*Gys*) was comparable among all groups (Fig. 4I). However, the genes related to glucose metabolism were affected by dietary L-carnitine supplementation. The mRNA level of *PFK*, a key glycolysis-related gene, was significantly decreased (fasting state) and increased (feeding state) in the muscle, but was comparable in the liver (Fig. 4B). The mRNA level of *PK*, another key glycolysis-related gene, was decreased in the muscle, and the decrease was significant in the feeding state (Fig. 4C). The mRNA expressions of *PECK1* and *G6Pa*, which are key gluconeogenesis-related genes, were significantly upregulated by dietary L-carnitine in the liver, but did not change in the muscle (Fig. 4D,E). The mRNA expressions of insulin sensitivity-related genes, including *insulin, Ira and Irb*, were mostly unaffected by dietary L-carnitine supplementation (Fig. 4F–H), except for *Irb* in the carnitine supplementation group, which was expressed at a much higher level in the liver in the feeding state (Fig. 4H). The interaction effects between the nutritional state and L-carnitine were only seen in the mRNA levels of *PFK* in muscle and *PECK1* and *Irb* mRNA levels in liver (Supplemental Table 4). In general, the above data suggested that dietary L-carnitine supplementation tended to increase gluconeogenesis, but decreased glycolysis; therefore, L-carnitine supplementation lowered the utilization of glucose.

### The effect of the dietary L-carnitine on proteins and genes of protein metabolism

**Figure 5:**
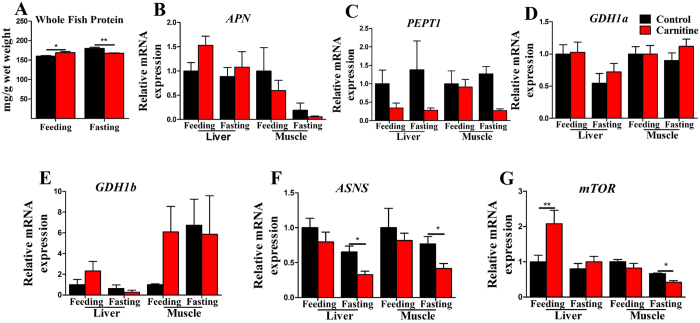
The effect of the dietary L-carnitine on protein and mRNA expression of the genes related to protein metabolism. All values are means ± SEM (n = 6). Values with *^,^**statistically differ at *P* < 0.05, *P* < 0.01.

To further understand the effect of dietary L-carnitine on the possible “protein-sparing effect” in zebrafish, the whole fish protein content and the mRNA expressions of genes related to protein metabolism were assayed. As shown in Fig. 5A, the crude protein level in the carnitine supplementation group was significantly increased compared with the control group in the feeding state, but was significantly decreased in the fasting state (Fig. 5A). The mRNA expressions of aminopeptidase N (*APN*) and oligopeptide transporter (*PEPT1*), which function in protein and amino acid digestion and absorption, and glutamate dehydrogenase 1a and 1b (*GDH1a, GDH1b*), which are essential in amino acid catabolism, were not affected significantly by dietary L-carnitine supplementation in the liver and muscle (Fig. 5B–E). The mRNA level of asparagine synthetase (*ASNS*), which plays roles in protein synthesis, was significantly decreased in the L-carnitine group in the fasting state, both the liver and muscle (Fig. 5F). Notably, in the L-carnitine supplementation group, the mRNA expression of *mTOR*, the regulatory factor for protein synthesis, was significantly increased in the liver in the feeding state (Fig. 5G). The interaction effects between the nutritional state and L-carnitine were observed in the whole fish protein content and the mRNA levels of *PEPT1* in the muscle (Supplemental Table 5). These data suggested that dietary L-carnitine supplementation tended to increase protein degradation by inhibiting protein synthesis in the fasting state. However, the increased crude protein levels in whole fish and the increased *mTOR* expression in the liver of carnitine-fed zebrafish also showed that L-carnitine has positive effects on protein synthesis in the normal feeding period.

### The effect of dietary L-carnitine on inflammatory gene expression

**Figure 6:**
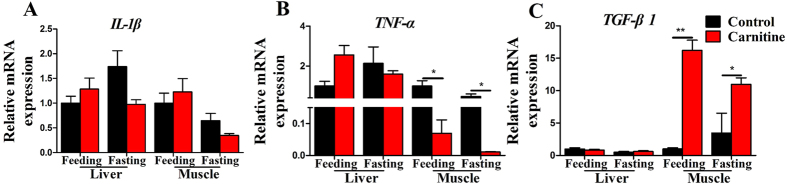
The effect of the dietary L-carnitine on mRNA expression of the genes related to inflammation. All values are means ± SEM (n = 6). Values with *^,^**statistically differ at *P* < 0.05, *P* < 0.01.

The expressions of three inflammation-related genes were measured to investigate the potential effects of dietary L-carnitine on inflammation. Among all groups, the mRNA expressions of *IL-1β* were comparable (Fig. 6A). The mRNA level of *TNF-α*, a strong inflammatory factor, was significantly decreased in the muscle of the L-carnitine supplementation group, in both the feeding and fasting states; however, no significant differences in the liver expression were found among the groups (Fig. 6B). The mRNA expression of *TGF-β1*, a known anti-inflammatory factor, was significantly increased by dietary L-carnitine supplementation in the muscle in the feeding and fasting states; however, its expression in the liver was not changed by L-carnitine supplementation (Fig. 6C). The interaction effects between the nutritional state and L-carnitine were observed in the mRNA levels of *IL-1β* and *TGF-β1* in liver and *TNF-α* in muscle (Supplemental Table 6). These data indicated that dietary L-carnitine is likely to play roles in inflammation processes.

## Discussion

As an essential factor in mitochondrial FA β-oxidation, L-carnitine has been used to alleviate fat accumulation-related metabolic syndromes in humans and other mammals for decades^8^,^9^,^30^. By contrast, in those fish that generally prefer to utilize protein, but not carbohydrate, to produce energy, carnitine has not only been used to decrease excess fat deposition in fish tissues, but is also thought to “save” the protein, which is used for energy through increasing the degradation of lipid for energy production. However, the effects of dietary carnitine supplementation in different fish species are contradictory^31^, but few previous studies performed comprehensive assays of lipid metabolism. In the present study, although growth promotion was not observed in zebrafish, dietary L-carnitine supplementation decreased the TG content in liver and muscle significantly, but did not affect the level in the viscera. Considering that liver and muscle, but not adipose tissue, are the main tissues for FA β-oxidation, the liver and muscle should be the main target tissues of dietary L-carnitine supplementation in fish. Indeed, the carnitine concentration in the liver and muscle of the experimental zebrafish increased with dietary L-carnitine supplementation.

By measuring the β-oxidation of [1-^14^C] palmitate in the mitochondria or peroxisome of the liver or muscle, the present study first indicated that dietary L-carnitine supplementation mainly increased mitochondrial FA β-oxidation activity, but not peroxisomal activity, at least in zebrafish. This was also confirmed by the high expression of *CPT1* mRNA in the liver and muscle of the L-carnitine-fed zebrafish, and the comparable mRNA expressions of ACO in both tissues between the groups. The present study also indicated that dietary L-carnitine supplementation downregulated significantly the expression of adipogenesis-related genes, such as ACC, FAS and DGAT. Therefore, the lipid-lowing effects of dietary L-carnitine supplementation in the present study are likely to be caused by increased mitochondrial FA β-oxidation activity and decreased lipid synthesis, which was in consistent with some mammalian studies^32^,^33^.

Mammalian and human studies showed that dyslipidaemia is accompanied frequently by inflammation^34^; however, the L-carnitine-induced upregulation of CPT1 has been shown to prevent inflammation by decreasing inflammatory cytokines, such as TNF-α, in serum and the liver^30^,^35^. Similarly, in the present study, L-carnitine supplementation significantly increased the expression of CPT1, accompanied by decreasing and increasing the mRNA expression of TNF-α and the anti-inflammatory factor TGF-β1, respectively, in zebrafish muscle. This is the first report of this anti-inflammatory effect of L-carnitine in fish. Lipid accumulation-related metabolic dysfunctions have been observed widely in aquatic animals; therefore, the anti-inflammatory effects of L-carnitine in fish require further study.

**Figure 7:**
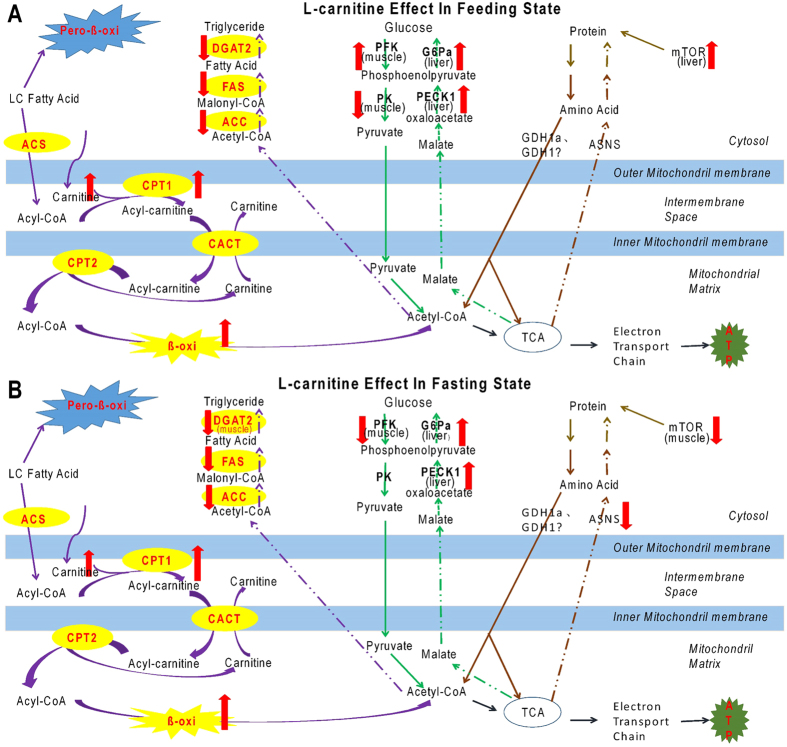
Systemic regulation of L-carnitine in nutritional metabolism of zebrafish. (**A**) The regulation of L-carnitine in feeding state. (**B**) The regulation of L-carnitine in fasting state. ACS, acyl-CoA synthetase. CACT, carnitine/acylcarnitine translocase.

Compared with mammals, fish have lower abilities to use carbohydrates as energy sources, thus fish cannot use glucose to produce energy efficiently^10^. A number of studies have indicated that, as compared with mammals, the responses of the activities of many glucose metabolism-related key enzymes, such as GK, PFK and PK, are not sensitive to the increased dietary carbohydrate content in fish^36^,^37^. In many fish species, high dietary carbohydrate induced lower growth, excess lipid deposition, and decreased stress resistance^38^,^39^,^40^. Therefore, fish nutritionists have tried different routes to promote the carbohydrate/glucose utilization in fish, including exploring the optimal sources and contents of dietary carbohydrate^41^,^42^, adding supplementary elements (e.g. chromium)^43^,^44^, or even developing gene-modified fish species^45^,^46^. However, in many fish species, these efforts are not ideal, and the regulatory mechanisms of carbohydrate metabolism are still poorly understood in fish. Recently, the interaction between lipid metabolism and carbohydrate metabolism has been observed in fish^47^,^48^. The phenomena that high-fat diet impairs glucose homeostasis has been reported in rainbow trout^49^,^50^. Our recent work further illustrated that glycolysis-related genes are upregulated or downregulated during low-fat diet or high-fat diet feeding, respectively^51^. In the present study, dietary L-carnitine supplementation improved lipid catabolism significantly, but increased the whole body glycogen deposition in the fasting state, indicating that glucose degradation was inhibited. This was also supported by the downregulated expressions of glycolysis-related genes (*PFK* and *PK*) in muscle, and the upregulated expressions in gluconeogenesis-related genes (*PECK1* and *G6Pa*) in the liver. Similarly, dietary L-carnitine supplementation also increased the pyruvate carboxylase (PC) activity and glucose production in the liver in Atlantic salmon^52^. This evidence indicated that in fish, increasing the catabolism of lipids decreases the energy portion sourced from glucose degradation, resulting in increased gluconeogenesis and glycogen synthesis. This could be explained if, during energy homeostasis in fish, the endogenous acetyl-CoA concentration is relatively stable; therefore, if the increased lipid catabolism produces more acetyl-CoA for energy production, the portion of the acetyl-CoA sourced from other nutrients, such as glucose, would decrease correspondingly. Thus, the glycolysis pathway would be downregulated and gluconeogenesis would be upregulated. The relationships of the metabolic pathways are illustrated in Fig. 7.

**Figure 8:**
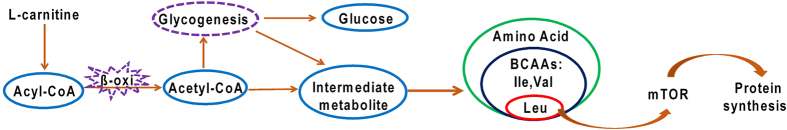
The possible mechanism of the L-carnitine on protein synthesis.

One of the concerns associated with the use of L-carnitine in fish feed is to increase energy supply from lipid catabolism and “save” the protein from energy production to increase protein deposition. A number of studies in different fish species have indicated that dietary L-carnitine reduced the lipid content and increased the protein content, or decreased the protein oxidation in tissues and/or the whole body, showing a significant protein-sparing effect^14^,^17^,^22^,^52^. However, the underlying molecular basis remained unknown. In the present study, dietary L-carnitine supplementation increased the protein content of the whole body in the feeding trial, and did not affect the mRNA expressions of genes associated with protein and amino acid catabolism. However, notably, dietary L-carnitine significantly increased the mRNA expression of *mTOR* in the liver in the feeding state. The mTOR protein is one of the most important regulatory elements of protein synthesis^53^; therefore, dietary supplementation with L-carnitine might have positive effects on protein synthesis in zebrafish. In Atlantic salmon, dietary L-carnitine supplementation increased the amino acid concentration in plasma and the liver, especially the three branched-chain amino acids, including leucine, isoleucine, and valine, and increased the protein synthesis capacity, accompanied by the accumulation of protein in organs^52^. The studies in mammals and rainbow trout reported that leucine can effectively stimulate mTOR signalling^54^,^55^. Thus, we deduced that dietary L-carnitine supplementation can stimulate the expression of mTOR in fish by increasing the leucine concentration in the feeding state. The specific mechanism of the protein sparing effect of L-carnitine is detailed in Fig. 8. However, the present study also indicated that dietary L-carnitine supplementation significantly decreased the whole body protein content in the fasting state, and the gene expressions of *ASNS* and *mTOR* in the L-carnitine groups were also downregulated in the muscle in the fasting state. This might be explained if L-carnitine accelerated the degradation of lipids and proteins in the fasting state, and inhibited the synthesis of lipids and proteins to larger degree compared with the control. Compared with the whole body glycogen content, which was increased in the L-carnitine supplementation group in the fasting state, the decreased lipid and protein content in L-carnitine supplementation group in the fasting state confirmed that fish prefer to utilize proteins and lipids rather than carbohydrates.

To illustrate the systemic regulation of nutritional metabolism by L-carnitine in zebrafish, the interaction of different metabolic pathways in the L-carnitine-fed fish is shown in Fig. 7. Briefly, in the feeding state, dietary L-carnitine supplementation can increase lipid catabolism to produce more energy by stimulating mitochondrial FA β-oxidation and reducing FA and TG synthesis, utilising the “protein sparing effect” to elevate protein synthesis. Meanwhile, dietary L-carnitine supplementation also inhibited glycolysis and enhanced gluconeogenesis to decrease glucose-derived energy production (Fig. 7A). In the fasting state, dietary L-carnitine supplementation had similar effects on lipid and glucose metabolism to the feeding state, but also increased protein degradation and reduced protein synthesis (Fig. 7B). Nevertheless, our results also indicated that the interaction effects between nutritional state and L-carnitine supplementation existed in many parameters, including nutrient compositions, biochemical activities and metabolism-related gene expressions. This indicates that the regulatory mechanisms of L-carnitine in feeding or fasting states as shown in Fig. 7A,B could not be understood separately. Furthermore, it is of note that the changes of gene mRNA expression do not directly reflect the enzyme activities or protein functions, therefore, the regulatory mechanisms of L-carnitine at the transcriptional level still need further functional validation.

## Conclusion

The present study indicated that dietary L-carnitine supplementation could increase the concentrations of carnitine in the liver and muscle, and decrease the lipid content in both tissues, possibly through enhanced mitochondrial β-oxidation and reduced adipogenesis. Dietary L-carnitine supplementation also increased the expression of mTOR in the liver, suggesting that it has positive roles in protein synthesis. However, dietary L-carnitine supplementation decreased glycolysis, because the increase in lipid-sourced ATP from L-carnitine supplementation might change the balance of energy homeostasis between lipids and carbohydrates. This is the first study to explore the mechanism of the positive effect of L-carnitine on the nutritional metabolism at transcriptional and biochemical levels in fish.

## Materials and Methods

### Animal ethics

All experiments were conducted strictly under the Guidance of the Care and Use of Laboratory Animals in China. This study was approved by the Committee on the Ethics of Animal Experiments of East China Normal University.

### Fish, Diets and Sampling

**Figure 9:**
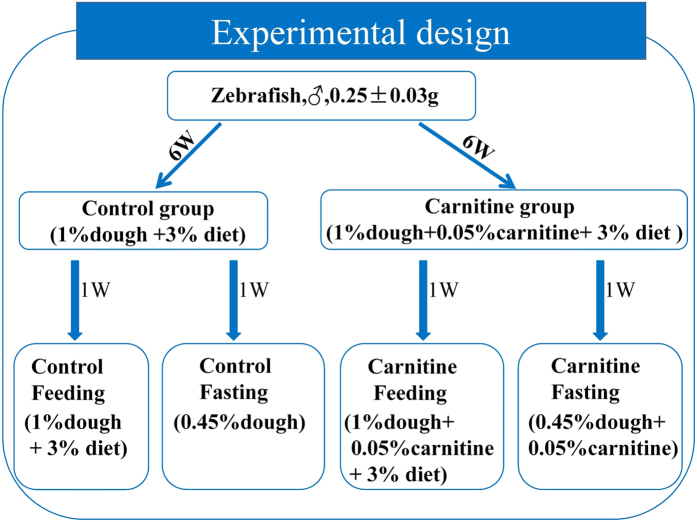
The design and the protocol of the present experiment.

In order to avoid the metabolic disturbance of estrogen during the sexual maturation of female fish, only male zebrafish (0.25 ± 0.03 g) were bought from Chinese National Zebrafish Resource Center (Wuhan, China). Before experiments, fish were acclimated in 100 cm × 45 cm × 45 cm tanks for 1 week and fed with commercial zebrafish diet (protein ≥ 50, lipid ≥ 8%) (Shengsuo Co., Shandong, China). After acclimation, six hundred zebrafish were randomly divided into 2 groups (3 tanks per group, 100 fish per tank): control group and carnitine group (Fig. 9). In the feeding trial, the carnitine was given by feeding fish with small wheat flour-dough particles containing L-carnitine (carnitine group) or not (control group), before basic diet feeding. In the preparation of the L-carnitine-contained wheat flour-dough particles, L-carnitine was first dissolved in pure water, and the carnitine solution was mixed with given amount of wheat flour to make wet dough. The wet dough was rubbed to small particles which size was proper for zebrafish, and then dried under 60 °C. When the dough particles were fed with 1% body weight, the L-carnitine composition in the dry wheat flour-dough particles could promise the final carnitine intake was 0.05% body weight, which is in the accordance with the dose used in other animals^56^,^57^. In the every morning, the dough particles containing L-carnitine or not were first fed to the carnitine group and control group, respectively, with 1% body weight. Afterwards, the same basic diet was fed to both groups with 3% body weight. The formulations of the basal diet and wheat flour-dough particle are listed in Table 1. The basal diet and dough particles were pelleted to a proper size (about 0.2 mg per particle) for zebrafish. After 6 weeks, the control and carnitine groups were divided into four groups: control-feeding, control-fasting, carnitine-feeding and carnitine-fasting (3 tanks/group and 50 fish/tank) (Fig. 9). In the two feeding groups, the feeding strategy was the same as that in the previous 6 weeks. In the two fasting groups, only dough particles were fed in the morning and the amount was decreased to 0.45% body weight, while the L-carnitine composition in the dough was increased to 10% to promise the same daily intake of L-carnitine with 0.05% body weight. Because the physiological effects of dietary L-carnitine supplementation were normally observed after 6–8 weeks in other animals^24^,^57^, the duration of the present experiment was 7 weeks. The experimental design was shown in Fig. 9. During the experiment, the photoperiod was 12 h:12 h, and the temperature was kept at 26 ± 2 °C. The weight of the fish in each tank was recorded every one week, and the feeding amount was adjusted correspondingly. At the end of the experiment, the whole fish in each tank were anthesthetized on ice, and sampled to collect liver, muscle and visceral for molecular and biochemical indexes. Hepatic, muscle and visceral triglyceride (TG) and whole fish glycogen were assessed by the commercial kit (Jiancheng Biotech Co., China). The crude lipid of the whole fish body was tested by using methanol and chloroform (1:2) as previously described^58^. Whole fish protein was measured by Kjeltec^TM^ 8200 (FOSS, Sweden). Because of the little mass of zebrafish organs (e.g. liver is 3–5 mg), samples were normally pooled from 2–4 fish. In the measurements and statistical calculations, 4–6 pooled samples were used (n = 4–6, see Figure legends).

### Carnitine concentration determination

After the feeding trial, the whole liver and parts of muscle of 4 fishes collected from each group were weighted and homogenized by pure-water (1:10, w/v) by using a drill-driven Teflon glass homogenizer (Scientz Biotec, China) with 3–4 strokes. Then the tissue homogenate centrifuged at 2000 rpm/min for 15 min (Centrifuge 5804R, Eppendorf, Germany). One hundred microliter aliquot of tissue homogenate supernatant was first incubated with 50 μL KOH (1 mol/L) at 37 °C for 30 min to fully hydrolyze the combined carnitine, and 10 μL HCl (1 mol/L) was then added to neutralize the solution (sample A). Sixty microliter distilled water was added directly into another 100 μL aliquot of tissue homogenate (sample B). Protein in sample A and B was removed by precipitating with 200 μL of cold ACN (containing 1 μg/mL carbachol, the internal standard, IS) and centrifuging at 16,900 × g for 20 min. One microliter of the supernatant was injected for LC-MS/MS analysis. Total carnitine was defined as the concentration of carnitine in sample A, free carnitine was defined as the concentration of carnitine in sample B.

An 1290 HPLC system (Agilent Technologies, Palo Alto, USA) and a 6460 triple-quadrupole mass spectrometer (Agilent Technologies, Palo Alto, USA) were used. The data was acquired and analyzed using MassHunter software (version 5.0.280.1; Agilent Technologies, Palo Alto, USA). Chromatographic separation was performed on a Phenomenex Luna 3u HILIC 200A Column (50*2 mm; Phenomenex, USA) due to the hydrophilicity and amphotericity of the analytes. The mobile phase system consisted of two phases, phase A (water) and phase B (water/acetonitrile, 1:9, v/v), and 10 mM of ammonium acetate was added to both of the phases. The analytes were separated on chromatographic column by isocratic elution (98% phase B) at the flow rate of 0.3 mL/min. The temperature of column oven was set at 40 °C, and the injection volume was 1 μL. The total run time for sample analysis was 4.5 min, and the flow was switched to MS during 0.8 to 3.1 min.

The mass spectrometer was operated in the positive electrospray ionization (ESI) mode, and the MS/MS analysis was performed in the multiple reaction monitoring (MRM) mode, monitoring the transitions of m/z 162.0 → 103.0 for carnitine, m/z 147.1 → 88.1 for IS, respectively. Fragmentation voltages/collision energy were optimized at 120 V/16 eV for carnitine, 90 V/14 eV for IS, respectively, for the best response of the transitions. Ion source conditions were set as follows: drying gas, 8 L/min at 300 °C; nebulizer gas, 20 psi; sheath gas, 9 L/min at 350 °C; collision gas (N2), 1.6 MPa; capillary voltage, 4000 V and nozzle voltage, 500 V.

### Mitochondrial and peroxisomal [1-^14^C] palmitate oxidation in liver and muscle

After the feeding trial, the whole liver and parts of muscle of 3 fish collected from each group were weighted and homogenized in the ice-cold 0.25M-sucrose medium containing 2 mM-EGTA and 10 mM-Tris-Cl, pH 7.4,(1:40 and 1:10, w/v) by using a drill-driven Teflon glass homogenizer with 3–4 strokes. The samples of homogenate were used for the immediate measurement of mitochondrial and peroxisomal [1-^14^C] palmitate β-oxidation. Palmitate oxidation rates were detected at 28 °C using two media as already described^59^, the first media allowing both mitochondrial and peroxisomal β-oxidation to occur, and the second one allowing peroxisomal β-oxidation only. After 0.5 h, the radio activity initially held by [1-^14^C] palmitate was recovered on labelled short molecules released from the β-oxidative cycle and soluble in perchloric acid (acid-soluble products, ASP). The pure radioactive ASP medium was collected using 0.45 μm membrane filters and measured after mixing with the scintillation cocktail described by Du *et al*.^60^ in a liquid scintillation spectrometer MicroBeta^2^ Plate Counter (Perkin, USA).

### Quantitative real-time PCR

Total RNA was isolated by using a Tri Pure Reagent (Aidlab, Cnina). The quality and quantity of total RNA were tested by NANODROP 2000 Spectrophpto (Thermo, USA). cDNAs of tissues total RNA were synthesized using a PrimerScript^TM^ RT reagent Kit with a gDNA Eraser (Perfect Real Time)(Takara, Japan) by S1000^TM^ Thermal Cycler (Bio-Rad, USA). Elongation factor 1 alpha (EF1α) and β-actin were used as the reference genes^61^. The primers of EF1α and target genes (Table 2) for Quentitative PCR (qPCR) were designed to overlap intron. qPCR (20 μL) was carried out by using 2 × Ultra SYBR Mixture (CWbio, China) in 12 × 8 well plates in a CFX Connect Real-Time System (Bio-Rad), containing 10 μL of SYBR Mixture, 2 μL cDNA, 1.6 μL of qPCR primers (4 μM), and 7.4 μL nuclease-free water. The program of qPCR reaction included 95 °C for 10 min, 40 cycles of 95 °C for 5 s and 60 °C for 15 s. The melting curves of amplified products were generated to ensure the specificity of assays at the end of each PCR. qPCR efficiency was between 98% and 102% and the correlation coefficient was over 0.97 for each gene. Each qPCR run performed in triplicate and negative controls (no cDNA) were conducted. The method of 2^−ΔΔCt^ was used for estimating the relative cDNA abundance (control feeding group as control).

### Statistical analyses

All results are presented as mean ± SEM. Independent-samples t-test was performed to evaluate the significant difference (P < 0.05) of variables between control and carnitine group in feeding or fasting states. Two-way ANOVA analysis was used to explore the possible interactions existing between nutritional states and L-carnitine supplementation in all parameters. All analyses were conducted using the SPSS Statistics 19.0 software (IBM, USA).

## Additional Information

**How to cite this article**: Li, J.-M. *et al*. Systemic regulation of L-carnitine in nutritional metabolism in zebrafish, *Danio rerio.* *Sci. Rep.* **7**, 40815; doi: 10.1038/srep40815 (2017).

**Publisher's note:** Springer Nature remains neutral with regard to jurisdictional claims in published maps and institutional affiliations.

## Supplementary Material

Supplementary Dataset

## Figures and Tables

**Table 1:** Formulation of the basic diet and wheat flour-dough particles.

Diet component (g/kg)	
Casein	280
Gelatin	70
Soybean	70
Corn starch	414.75
Vitamin premix^1^	15
Mineral premix^2^	45
CMC	30
Cellulose	70
Choline chloride	5
BHT	0.25
Total protein	350
Total lipid	70
Total carbohydrate	414.75
Nutrient composition	
Dry matter (%)	92.32
Crude protein (%)	34.59
Crude lipid (%)	6.89
Wheat flour component (100 g)	
Protein (g)	12.2
Lipid (g)	1.5
Carbohydrate (g)	70

^1^Vitamin premix, (mg or IU/kg): 500,000 I.U. (international units) Vitamin A, 50,000 I.U. Vitamin D3, 2500 mg Vitamin E, 1000 mg Vitamin K3, 5000 mg Vitamin B1, 5000 mg Vitamin B2, 5000 mg Vitamin B6, 5000 μg Vitamin B12, 25,000 mg Inositol, 10,000 mg Pantothenic acid, 100,000 mg Cholin, 25,000 mg Niacin, 1000 mg Folic acid, 250 mg Biotin, 10,000 mg Vitamin C.

^2^Mineral premix, (g/kg): 314.0 g CaCO_3_; 469.3 KH_2_PO_4_; 147.4 g MgSO_4_·7H_2_O; 49.8 g NaCl; 10.9 g Fe (II) gluconate; 3.12 g MnSO_4_·H_2_O; 4.67 g ZnSO_4_·7H_2_O; 0.62 g CuSO_4_·5H_2_O; 0.16 g KJ; 0.08 g CoCl_2_·6H_2_O; 0.06 g NH_4_ molybdate; 0.02 g NaSeO_3_.

**Table 2:** The primers used in the experiment.

Gene name	Primers (5′-3′)	GenBank NO.
Elongation factor 1 α (EF1α)	F: CCCCTGGACACAGAGACTTCATC	L23807.1
	R: ATACCAGCCTCAAACTCACCGAC	
β-actin	F: TCTGGTGATGGTGTGACCCA	AY222742
	R: GGTGAAGCTGTAGCCACGCT	
gamma-butyrobetaine hydroxylase 1 (BBOX1)	F: CCCATGGCTAACAATGTTGCCTA	NM_001017717.1
	R: ATCAGCCTGACGGACACAATGTA	
Carnitine palmitoyltransferase 1 (CPT1, liver)	F: CATCCTTAGGCCTGCTCTTCAAA	NM_001044854
	R: ACCATGACACCCCCAACTAACAT	
Carnitine palmitoyltransferase 1 (CPT1, muscle)	F: CCTCCATGGGCACGATTGATAA	NM_001005940.1
	R: GCAAACAGGATGGCACTCAACA	
Enoyl-CoA, hydratase/3-hydroxyacyl CoA dehydrogenase (HAD)	F: GAATACTTGTGAGGTGGCTCTGGA	NM_207068.1
	R: AGGACACGGTGTGGTCAGCAT	
Acyl-CoA oxidase 3 (ACOX3)	F: TGGAAGGACATGATGCGCTTT	NM_213147.1
	R: AGGCTGCCGGGCAAAAA	
Acetyl-CoA carboxylase (ACC)	F: GCGTGGCCGAACAATGGCAG	NM_001271308.1
	R: GCAGGTCCAGCTTCCCTGCG	
Fatty acid synthase (FAS)	F: GGAGCAGGCTGCCTCTGTGC	XM_009306806.1
	R: TTGCGGCCTGTCCCACTCCT	
Diacylglycerol O-acyltransferase 2 (DGAT2)	F: ACGCATAACCTGCTTCCC	NM_001030196.1
	R: TCCTGTGGCTTCTGTCCC	
Lipoprotein lipase (LPL)	F: ACATTTCCTCGGGATTGGAAACT	NM_131127.1
	R: TCCATCATCCATTCTGTGGCAT	
Cluster determinant (CD36)	F: TGAACAAAATCAAGGAGCACACAA	NM_001002363.1
	R: ATCCGGGAAATCAGCTCATTCTT	
Adipose triglyceride lipase (ATGL)	F: GCGTGACGGATGGAGAAA	KP325485.1
	R: AGGCCACAGTAAACAGGAATAT	
Hormone-sensitive lipase (HSL)	F: CGGCAAGGACAGGACAGT	NM_001316725.1
	R: GCATGGAGAAAGAGGAGCT	
Phosphofructokinase (PFK, liver)	F: GTAACACGCATGGGCATTTTTG	NM_001017596
	R: TCGCCAGTTTGATGTGATCTCCT	
Phosphofructokinase (PFK, muscle)	F: ATCACATCCGTCCTGCTACATGG	NM_00100457
	R: TGGTCTGGAAATCCTTACAGCG	
Pyruvate kinase (PK, liver)	F: ATCACTGCCCGCAACACCA	NM_201289.1
	R: TCATTCCTGCTTTCACCATCTCC	
Pyruvate kinase (PK, muscle)	F: TGAACATCGCTCGCATGAACTT	NM_199333.1
	R: TCAAAGCTGGCACAAGCTTCA	
Glucose-6-phosphatase (G6Pa)	F: TGGCAGTGATAGGAGATTGGCTT	BC148168.1
	R: AGTAGGACGTCTCATGGACCCAC	
Phosphoenolpyruvate carboxykinase 1 (PECK1)	F: ATCGCATCACGCATCGCTAAA	NM_214751
	R: CCGCTGCGAAATACTTCTTCTGT	
Insulin	F: ACAGGCTTCTTCTACAACCCCAA	AJ237750
	R: AAATGCAAAGTCAGCCACCTCA	
Insulin receptor a (Ira)	F: TTGTGATGGAGGGAGGATATCTGG	NM_001142672.1
	R: GGGCCGCATTTTGGGATTAT	
Insulin receptor b (Irb)	F: TTTCGCCTACATCTTGTGCCTCT	NM_001123229.1
	R: AGTTCTCCAAAACCCGCAGGTT	
Glycogen synthase 1 (Gys1, muscle)	F: GGCACTCAGGAGAACCATTGATAA	NM_201180.1
	R: TCCAGCAGAACCACATATGGTGA	
Glycogen synthase 2 (Gys2, liver)	F: TTGAAGATCTCCTGCTCTTTGAGG	NM_001018679.1
	R: CATTCGTCCACAGTGATCTTTGCT	
Aminopeptidase n (APN)	F: GGTGGCTTTTACCGGAGTGAATA	XM_001920383.5
	R: CAAGGAAATGCTTTTCTGGCATC	
Peptide transporter 1 (PEPT1)	F: TGGTGAATGAGTTCTGTGAGCGA	AY300011.1
	R: ACAGGTCATCATCCCAACCAATG	
Glutamate dehydrogenase 1a (GDH1a)	F: AGGACATTGTGCATTCGGGATT	NM_212576
	R: CCTCAGATCCAGCCCAAGGTTAT	
Glutamate dehydrogenase 1b (GDH1b)	F: GATGTCCTGGATTGCTGACACCT	NM_199545
	R: CCACCCTGGCTAATGGGTTTT	
Asparagine synthetase (ASNS)	F: TTCAGAATGCTGACTGACGATGG	NM_20116
	R: TGGAAAAGCAGTGATCTTTGCAG	
mechanistic target of rapamycin (mTOR)	F: TGGGAGCAGACAGGAATGAAGG	NM_001077211.2
	R: TGCACCTGCTGGAAAAAGAATG	
Interleukin 1 beta (IL-1β)	F: ATGATGGCATGCGGGCAATAT	NM_212844.2
	R: AGCGGATCTGAACAGTCCATCTC	
Transforming growth factor β1 (TGF-β1)	F: ACTACTTTGGCAAGGAGGTGCAT	NM_182873.1
	R: CATCTCGGACACGTTGAAAAACA	
Tumor necrosis factor a (TNF-α)	F: TCTGCTTCACGCTCCATAAGACC	NM_212859.2
	R: GCCTTGGAAGTGAAATTGCCTT	
