# Corrigendum: Systemic regulation of L-carnitine in nutritional metabolism in zebrafish, *Danio* *rerio*

**DOI:** 10.1038/srep44970

**Published:** 2017-03-23

**Authors:** Jia-Min Li, Ling-Yu Li, Xuan Qin, Li-Jun Ning, Dong-Liang Lu, Dong-Liang Li, Mei-Ling Zhang, Xin Wang, Zhen-Yu Du

Scientific Reports 7: Article number: 4081510.1038/srep40815; published online: 01 19 2017; updated: 03 23 2017

The original version of this Article contained an error in the spelling of the author Xuan Qin, which was incorrectly given as Xun Qin. This has now been corrected in the PDF and HTML versions of the Article.

