# Supplementary material for: Systemic regulation of L-carnitine in nutritional metabolism in zebrafish, Danio rerio
Source: Sci Rep. 2017 Jan 19;7:40815. doi: 10.1038/srep40815 (PMC5244368; doi:10.1038/srep40815)
Supplement: Supplementary Dataset [file srep40815-s1.doc]

**Systemic regulation of L-carnitine in nutritional metabolism in zebrafish, *Danio rerio***

Jia-Min Li^a^, Ling-Yu Li^a^, Xun Qin^b^, Li-Jun Ning^a^, Dong-Liang Lu^a^, Dong-Liang Li^a^, Mei-Ling Zhang^a^, Xin Wang^b*^, Zhen-Yu Du^a*^

^a^ Laboratory of Aquaculture Nutrition and Environmental Health (LANEH), School of Life Sciences, East China Normal University, Shanghai, China

^b^Shanghai Key Laboratory of Regulatory Biology, Institute of Biomedical Sciences and School of Life Sciences, East China Normal University, Shanghai, China

**Corresponding authors:**

^*^Prof. Zhen-Yu Du

Laboratory of Aquaculture Nutrition and Environmental Health, School of Life Sciences, East China Normal University, Shanghai 200241, PR China

E-mail address: [zydu@bio.ecnu.edu.cn](mailto:zydu@bio.ecnu.edu.cn);

Telephone: + 86-21-54345354

Dr. Xin Wang

Shanghai Key Laboratory of Regulatory Biology, Institute of Biomedical Sciences and School of Life Sciences, East China Normal University, Shanghai, China

E-mail address: [xwang@bio.ecnu.edu.cn](mailto:xwang@bio.ecnu.edu.cn)

| **Supplemental Table 1 The statistical calculation of the values in Figure 1** | | | | | | | |
| --- | --- | --- | --- | --- | --- | --- | --- |
|  | | One-way ANOVA |  | Two-way ANOVA | | | |
|  |  | Significance  (*P* value) |  |  | Nutritional state (N) | Carnitine (C) | N×C |
| Fig.2A  Free carnitine | Liver | 0.001 |  | *F* | 14.115 | 68.695 | 3.102 |
|  |  |  |  | *P* | 0.009 | 0.000 | 0.129 |
|  | Muscle | 0.005 |  | *F* | 3.500 | 22.694 | 3.256 |
|  |  |  |  | *P* | 0.098 | 0.001 | 0.109 |
| Fig.2B  Total carnitine | Liver | 0.000 |  | *F* | 0.647 | 100.120 | 2.354 |
|  |  |  |  | *P* | 0.445 | 0.000 | 0.163 |
|  | Muscle | 0.000 |  | *F* | 0.700 | 90.646 | 0.494 |
|  |  |  |  | *P* | 0.427 | 0.000 | 0.502 |
| Fig.2C  *BBOX1* | Liver | 0.023 |  | *F* | 5.978 | 0.003 | 5.023 |
|  |  |  |  | *P* | 0.025 | 0.955 | 0.038 |
|  | Muscle | 0.005 |  | *F* | 11.576 | 0.098 | 9.784 |
|  |  |  |  | *P* | 0.003 | 0.758 | 0.006 |
| Fig.2D  Whole fish lipid content |  | 0.184 |  | *F* | 4.896 | 0.077 | 0.358 |
|  |  |  |  | *P* | 0.039 | 0.784 | 0.556 |
| Fig.2E  Triglyceride content | Liver | 0.004 |  | *F* | 0.775 | 17.219 | 0.209 |
|  |  |  |  | *P* | 0.390 | 0.001 | 0.653 |
|  | Muscle | 0.000 |  | *F* | 14.755 | 13.602 | 0.775 |
|  |  |  |  | *P* | 0.001 | 0.002 | 0.390 |
|  | Viseral | 0.345 |  | *F* | 0.014 | 3.283 | 0.224 |
|  |  |  |  | *P* | 0.908 | 0.085 | 0.641 |

| **Supplemental Table 2 The statistical calculation of the values in Figure 2** | | | | | | |
| --- | --- | --- | --- | --- | --- | --- |
|  | | One-way ANOVA | Two-way ANOVA | | | |
|  |  | Significance  (*P* value) |  | Nutritional state (N) | Carnitine (C) | N×C |
| Fig.3A  Mitochondrial β-oxidation | Liver | 0.001 | *F* | 1.919 | 53.996 | 0.390 |
|  |  |  | *P* | 0.203 | 0.000 | 0.549 |
|  | Muscle | 0.007 | *F* | 0.264 | 1.556 | 28.875 |
|  |  |  | *P* | 0.626 | 0.259 | 0.002 |
| Fig.3B  Peroxisomal β-oxidation | Liver | 0.738 | *F* | 0.298 | 0.006 | 0.984 |
|  |  |  | *P* | 0.600 | 0.943 | 0.350 |
|  | Muscle | 0.276 | *F* | 0.732 | 0.590 | 3.315 |
|  |  |  | *P* | 0.417 | 0.465 | 0.106 |
| Fig.3C  Total β-oxidation | Liver | 0.001 | *F* | 1.863 | 42.086 | 0.664 |
|  |  |  | *P* | 0.209 | 0.000 | 0.439 |
|  | Muscle | 0.022 | *F* | 0.256 | 2.000 | 15.138 |
|  |  |  | *P* | 0.628 | 0.200 | 0.006 |

| **Supplemental Table 3 The statistical calculation of the values in Figure 3** | | | | | | |
| --- | --- | --- | --- | --- | --- | --- |
|  | | One-way ANOVA | Two-way ANOVA | | | |
|  |  | Significance  (*P* value) |  | Nutritional state  (N) | Carnitine  (C) | N×C |
| Fig.4A  *CPT1* | Liver | 0.001 | *F* | 6.840 | 17.488 | 0.991 |
|  |  |  | *P* | 0.017 | 0.000 | 0.331 |
|  | Muscle | 0.010 | *F* | 0.098 | 15.542 | 0.277 |
|  |  |  | *P* | 0.758 | 0.001 | 0.606 |
| Fig.4B  *HAD* | Liver | 0.276 | *F* | 3.163 | 0.370 | 0.627 |
|  |  |  | *P* | 0.091 | 0.550 | 0.438 |
|  | Muscle | 0.626 | *F* | 0.854 | 0.398 | 0.479 |
|  |  |  | *P* | 0.367 | 0.535 | 0.497 |
| Fig.4C  *ACOX3* | Liver | 0.000 | *F* | 47.927 | 0.103 | 0.378 |
|  |  |  | *P* | 0.000 | 0.752 | 0.546 |
|  | Muscle | 0.171 | *F* | 1.612 | 0.638 | 3.287 |
|  |  |  | *P* | 0.219 | 0.434 | 0.085 |
| Fig.4D  *ACC* | Liver | 0.000 | *F* | 47.322 | 13.093 | 10.681 |
|  |  |  | *P* | 0.000 | 0.002 | 0.004 |
|  | Muscle | 0.000 | *F* | 26.934 | 39.448 | 14.602 |
|  |  |  | *P* | 0.000 | 0.000 | 0.001 |
| Fig.4E  *FAS* | Liver | 0.000 | *F* | 49.621 | 35.584 | 34.964 |
|  |  |  | *P* | 0.000 | 0.000 | 0.000 |
|  | Muscle | 0.000 | *F* | 12.483 | 19.712 | 14.310 |
|  |  |  | *P* | 0.003 | 0.000 | 0.002 |
| Fig.4F  *DGAT2* | Liver | 0.000 | *F* | 22.101 | 8.970 | 11.637 |
|  |  |  | *P* | 0.000 | 0.007 | 0.003 |
|  | Muscle | 0.000 | *F* | 23.530 | 21.642 | 13.471 |
|  |  |  | *P* | 0.000 | 0.000 | 0.002 |
| Fig.4G  *LPL* | Liver | 0.000 | *F* | 42.681 | 1.927 | 7.986 |
|  |  |  | *P* | 0.000 | 0.180 | 0.010 |
|  | Muscle | 0.019 | *F* | 2.973 | 7.488 | 2.156 |
|  |  |  | *P* | 0.101 | 0.013 | 0.158 |
| Fig.4H  *CD36* | Liver | 0.011 | *F* | 8.969 | 2.693 | 3.144 |
|  |  |  | *P* | 0.007 | 0.117 | 0.092 |
|  | Muscle | 0.369 | *F* | 0.471 | 1.597 | 1.278 |
|  |  |  | *P* | 0.501 | 0.222 | 0.273 |
| Fig.4I  *ATGL* |  | 0.010 | *F* | 4.358 | 5.646 | 5.086 |
|  |  |  | *P* | 0.051 | 0.029 | 0.037 |
| Fig.4J  *HSL* |  | 0.000 | *F* | 41.259 | 0.060 | 0.308 |
|  |  |  | *P* | 0.000 | 0.808 | 0.585 |

| **Supplemental Table 4 The statistical calculation of the values in Figure 4** | | | | | | |
| --- | --- | --- | --- | --- | --- | --- |
|  | | One-way ANOVA | Two-way ANOVA | | | |
|  |  | Significance  (*P* value) |  | Nutritional state (N) | Carnitine (C) | N×C |
| Fig.5A  Whole fish glycogen content |  | 0.003 | *F* | 16.638 | 1.880 | 0.998 |
|  |  |  | *P* | 0.001 | 0.186 | 0.330 |
| Fig.5B  *PFK* | Liver | 0.519 | *F* | 0.946 | 0.028 | 1.363 |
|  |  |  | *P* | 0.342 | 0.869 | 0.257 |
|  | Muscle | 0.023 | *F* | 0.414 | 0.002 | 11.962 |
|  |  |  | *P* | 0.529 | 0.964 | 0.003 |
| Fig.5C  *PK* | Liver | 0.000 | *F* | 46.503 | 0.157 | 0.016 |
|  |  |  | *P* | 0.000 | 0.696 | 0.900 |
|  | Muscle | 0.008 | *F* | 3.710 | 10.633 | 2.377 |
|  |  |  | *P* | 0.071 | 0.005 | 0.142 |
| Fig.5D  *PECK1* | Liver | 0.000 | *F* | 47.567 | 22.795 | 10.799 |
|  |  |  | *P* | 0.000 | 0.000 | 0.004 |
|  | Muscle | 0.169 | *F* | 5.360 | 0.284 | 0.132 |
|  |  |  | *P* | 0.035 | 0.602 | 0.722 |
| Fig.5E  *G6Pa* | Liver | 0.000 | *F* | 51.152 | 13.574 | 2.800 |
|  |  |  | *P* | 0.000 | 0.001 | 0.110 |
|  | Muscle | 0.048 | *F* | 6.317 | 0.969 | 2.235 |
|  |  |  | *P* | 0.021 | 0.337 | 0.151 |
| Fig.5F  *Insulin* | Liver | 0.136 | *F* | 2.178 | 2.477 | 1.599 |
|  |  |  | *P* | 0.156 | 0.132 | 0.221 |
|  | Muscle | 0.468 | *F* | 1.516 | 0.001 | 0.476 |
|  |  |  | *P* | 0.236 | 0.978 | 0.500 |
| Fig.5G  *Ira* | Liver | 0.581 | *F* | 1.092 | 0.617 | 0.298 |
|  |  |  | *P* | 0.308 | 0.441 | 0.591 |
|  | Muscle | 0.005 | *F* | 13.597 | 1.533 | 2.023 |
|  |  |  | *P* | 0.001 | 0.230 | 0.170 |
| Fig.5H  *Irb* | Liver | 0.000 | *F* | 33.868 | 3.137 | 7.256 |
|  |  |  | *P* | 0.000 | 0.092 | 0.014 |
|  | Muscle | 0.444 | *F* | 1.043 | 0.071 | 1.677 |
|  |  |  | *P* | 0.319 | 0.792 | 0.210 |
| Fig.5I  *Gys* | Liver | 0.138 | *F* | 4.782 | 1.042 | 0.355 |
|  |  |  | *P* | 0.041 | 0.320 | 0.558 |
|  | Muscle | 0.252 | *F* | 0.273 | 3.794 | 0.375 |
|  |  |  | *P* | 0.607 | 0.066 | 0.548 |

| **Supplemental Table 5 The statistical calculation of the values in Figure 5** | | | | | | |
| --- | --- | --- | --- | --- | --- | --- |
|  | | One-way ANOVA |  | Two-way ANOVA | | |
|  |  | Significance  (*P* value) |  | Nutritional state  (N) | Carnitine  (C) | N×C |
| Fig.6A  Whole fish protein  content |  | 0.000 | *F* | 15.669 | 0.624 | 21.111 |
|  |  |  | *P* | 0.001 | 0.439 | 0.000 |
| Fig.6B  *APN* | Liver | 0.230 | *F* | 1.557 | 2.567 | 0.556 |
|  |  |  | *P* | 0.227 | 0.125 | 0.464 |
|  | Muscle | 0.188 | *F* | 2.836 | 1.358 | 0.299 |
|  |  |  | *P* | 0.120 | 0.268 | 0.596 |
| Fig.6C  *PEPT1* | Liver | 0.182 | *F* | 0.164 | 5.112 | 0.327 |
|  |  |  | *P* | 0.691 | 0.039 | 0.576 |
|  | Muscle | 0.009 | *F* | 0.879 | 7.546 | 5.295 |
|  |  |  | *P* | 0.362 | 0.014 | 0.035 |
| Fig.6D  *GDH1a* | Liver | 0.092 | *F* | 6.670 | 0.450 | 0.259 |
|  |  |  | *P* | 0.018 | 0.510 | 0.617 |
|  | Muscle | 0.636 | *F* | 0.003 | 0.813 | 0.799 |
|  |  |  | *P* | 0.954 | 0.380 | 0.384 |
| Fig.6E  *GDH1b* | Liver | 0.078 | *F* | 4.776 | 0.787 | 2.345 |
|  |  |  | *P* | 0.041 | 0.386 | 0.141 |
|  | Muscle | 0.557 | *F* | 0.922 | 0.540 | 1.086 |
|  |  |  | *P* | 0.350 | 0.473 | 0.312 |
| Fig.6F  *ASNS* | Liver | 0.003 | *F* | 13.724 | 5.736 | 0.304 |
|  |  |  | *P* | 0.001 | 0.027 | 0.588 |
|  | Muscle | 0.050 | *F* | 5.269 | 3.744 | 0.370 |
|  |  |  | *P* | 0.035 | 0.070 | 0.551 |
| Fig.6G  *mTOR* | Liver | 0.005 | *F* | 7.172 | 7.155 | 3.404 |
|  |  |  | *P* | 0.014 | 0.015 | 0.080 |
|  | Muscle | 0.001 | *F* | 18.151 | 5.841 | 0.152 |
|  |  |  | *P* | 0.001 | 0.027 | 0.702 |

| **Supplemental Table 6 The statistical calculation of the values in Figure 6** | | | | | | |
| --- | --- | --- | --- | --- | --- | --- |
|  | | One-way ANOVA | Two-way ANOVA | | | |
|  |  | Significance  (*P* value) |  | Nutritional state  (N) | Carnitine  (C) | N×C |
| Fig.7A  *IL-1β* | Liver | 0.069 | *F* | 1.020 | 1.266 | 6.014 |
|  |  |  | *P* | 0.325 | 0.274 | 0.024 |
|  | Muscle | 0.014 | *F* | 10.627 | 0.032 | 1.919 |
|  |  |  | *P* | 0.005 | 0.860 | 0.184 |
| Fig.7B  *TNF-α* | Liver | 0.130 | *F* | 6.831 | 0.012 | 0.976 |
|  |  |  | *P* | 0.018 | 0.914 | 0.336 |
|  | Muscle | 0.000 | *F* | 0.779 | 52.925 | 6.115 |
|  |  |  | *P* | 0.391 | 0.000 | 0.026 |
| Fig.7C  *TGF-β1* | Liver | 0.070 | *F* | 0.035 | 1.147 | 4.788 |
|  |  |  | *P* | 0.854 | 0.298 | 0.042 |
|  | Muscle | 0.000 | *F* | 5.684 | 31.842 | 3.644 |
|  |  |  | *P* | 0.030 | 0.000 | 0.074 |
